# Connected iridium nanoparticle catalysts coated onto silica with high density for oxygen evolution in polymer electrolyte water electrolysis†

**DOI:** 10.1039/c9na00568d

**Published:** 2019-12-02

**Authors:** Yoshiyuki Sugita, Takanori Tamaki, Hidenori Kuroki, Takeo Yamaguchi

**Affiliations:** Laboratory for Chemistry and Life Science, Tokyo Institute of Technology R1-17, 4259 Nagatsuta, Midori-ku Yokohama 226-8503 Japan yamag@res.titech.ac.jp; Kanagawa Institute of Industrial Science and Technology R1-17, 4259 Nagatsuta, Midori-ku Yokohama 226-8503 Japan

## Abstract

We propose connected Ir nanoparticle catalysts (Ir/SiO_2_) by coating 1.8 nm Ir particles with high density onto silica for the oxygen evolution reaction. Nanoparticles form electron-conducting networks, which can eliminate the need for an electron-conducting support. Ir/SiO_2_ showed a high electrochemical surface area, mass activity, and water electrolysis performance.

## Abstract 2

Connected Ir nanoparticle catalysts without any electron-conducting support are prepared for oxygen evolution reaction in polymer electrolyte water electrolysis.

## Graphical abstract

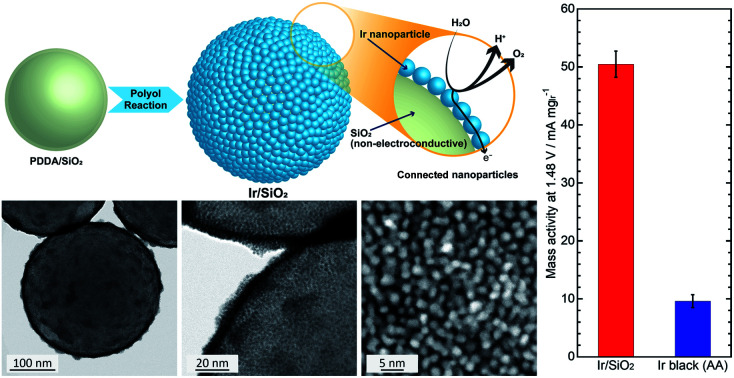

Water electrolysis has attracted increasing attention as a method for converting electricity generated by renewable energy to hydrogen (power to gas), especially because of the rapid decrease in the cost of renewable energy around the world.^1^ The energy conversion requires responsiveness to varying loads due to an uneven temporal distribution of renewable energy. Although alkaline liquid electrolyte water electrolysis has operated at the plant-scale, we focus on polymer electrolyte water electrolysis (PEWE), which is suitable for hydrogen production for renewable energy due to rapid system response times and higher efficiencies as a result of lower ohmic losses.^2–4^ These advantages come from commercially available solid proton-conducting membranes such as perfluorosulfonic acid polymers like Nafion®. Moreover, PEWE has some additional advantages such as lower gas permeability, production of high-purity and pressurized hydrogen without a pump, and a supply of neutral water. However, PEWE requires the use of precious metals as catalysts because non-precious metals are dissolved in the acidic conditions. The high cost owing to the large amounts of precious metals is one of the most important issues to be solved in PEWE. Specifically, anode catalysts for the oxygen evolution reaction (OER) have been a topic of multiple studies because the OER kinetics are slower than the hydrogen evolution reaction kinetics in the cathode.^5^ Iridium (Ir) and ruthenium (Ru) have often been reported as anode catalysts for PEWE^6–8^ because of their low OER overpotential. Ir is more suitable for the PEWE anode because Ru has poor stability at highly oxidizing potentials. Ir-based alloy and core–shell catalysts^9–13^ have succeeded in achieving high activity and stability. Despite these advancements, the OER for PEWE still requires a high Ir loading of 1–4 mg cm^−2^ mainly due to the lack of supports used for the catalysts as discussed below. In general, electrode catalysts in electrochemical cells, such as fuel cells, use electron-conducting supports, such as carbon, to maintain a high surface area. However, a carbon support cannot be used for a PEWE anode where a highly oxidizing potential is applied and most supports including carbon are corroded. Thus, low surface area Ir catalysts are used, which requires the high Ir loading. To reduce the precious metal loading and solve the issues of electron-conducting supports, nanocatalysts supported on metal oxide,^14–16^ support-free porous catalysts,^17–20^ and nanowires^21^ have been reported. Metal oxide supports are relatively stable in operation at high potential compared to carbon. Nevertheless, some problems exist, such as decreased performance due to passivation caused by metal oxidation, catalyst detachment from supports,^22^ and decreased proton conductivity due to the exchange of the cation in the ionomer to the titanium cation formed in operation at more than 1 A cm^−2^.^23^ Support-free catalysts, such as porous catalysts and nanowires,^17–21^ can essentially form a catalyst layer without conducting support materials, while some reports have used supports for the evaluation of the OER performance of catalysts,^17–19^ and some membrane electrode assembly (MEA) without any supports use a high Ir loading of 2.0 mg cm^−2^.^20^

In the present study, we suggest the use of connected Ir nanoparticle catalysts showing electron conductivity and a high surface area without any conducting supports. The electron-conducting support can be eliminated due to the formation of electron-conducting networks by the coating of Ir nanoparticles onto silica templates with high density. Because silica does not have an electron conductivity, corrosion/dissolution of silica does not affect the performance of the catalyst. Transmission electron microscopy (TEM) observations suggests that the networks are retained to some extent without aggregation even after dissolution of the silica template. Fig. 1(a) shows a schematic image of connected Ir nanoparticle catalysts and electron conduction through the nanoparticles.

**Fig. 1:**
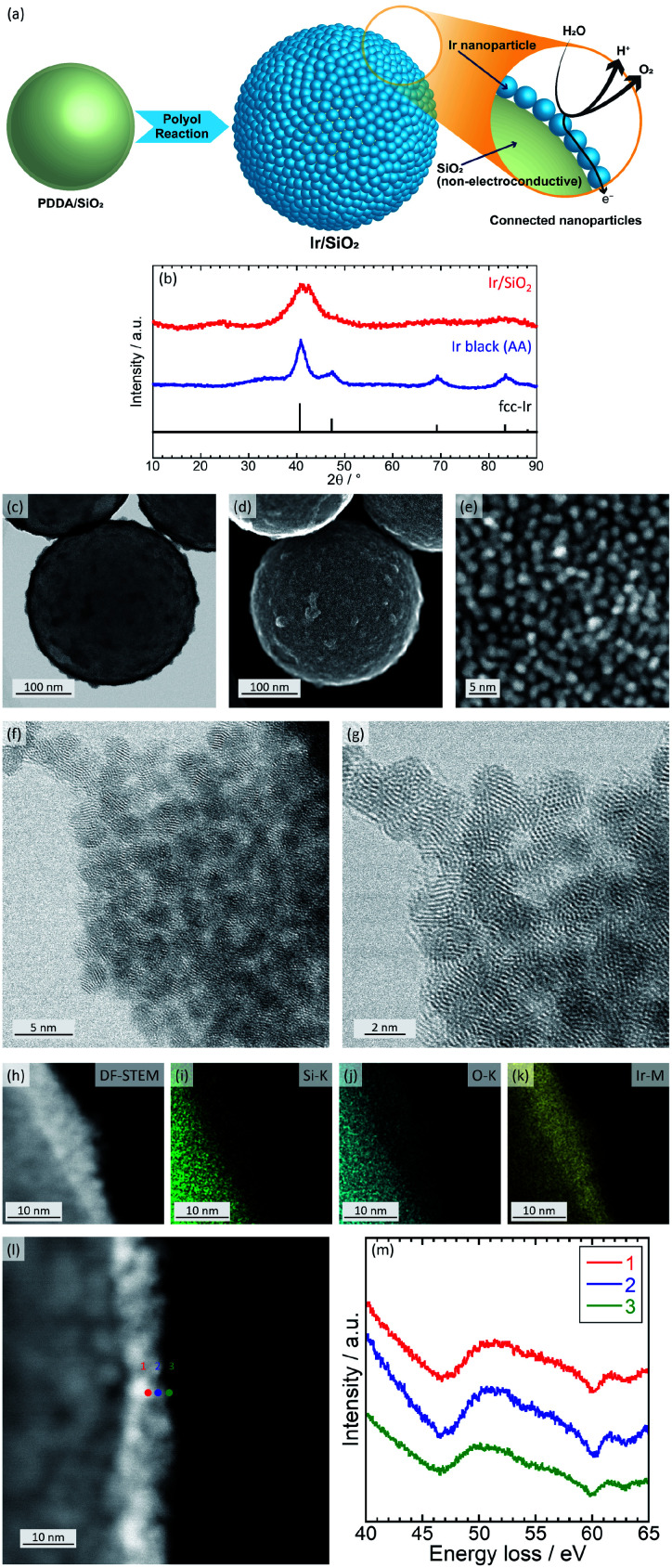
(a) Schematic image of connected Ir nanoparticle catalysts and electron conduction through the nanoparticles. (b) XRD patterns for the Ir catalysts. The peak data for fcc-Ir was obtained from a database (ICSD no. 64992). (c) BF-STEM and (d) and (e) SE-STEM images of Ir/SiO_2_ obtained using HD-2700. (f) and (g) BF-STEM of Ir/SiO_2_ nanoparticles. (h) DF-STEM, (i) Si–K, (j) O–K and (k) Ir–M images for EDX mapping of Ir/SiO_2_. (l) DF-STEM and (m) energy loss for EELS analysis of Ir/SiO_2_. (f)–(m) Data was obtained using HF5000 (Hitachi High-Technologies).

The catalysts were prepared in a similar way to connected platinum–iron nanoparticle catalysts;^24,25^ silica spheres with a diameter of 300 nm are first coated by poly(diallyldimethylammonium chloride); then, Ir nanoparticles are formed on the surface-modified silica with high density. For comparison, commercially available Ir black (Alfa Aesar (AA)) was used as a catalyst. Catalysts were characterized with inductively coupled plasma atomic emission spectroscopy (ICP-AES), X-ray diffraction (XRD), scanning transmission electron microscopy (STEM), energy dispersive X-ray spectrometry (EDX), electron energy loss spectroscopy (EELS), and X-ray photoelectron spectroscopy (XPS). The STEM (Fig. 1(f) and (g)), EDX (Fig. 1(h)–(k)), and EELS (Fig. 1(l) and (m)) analyses were measured using the field emission transmission electron microscope HF5000 (Hitachi High-Technologies). Details of the synthesis and structural characterization are described in the ESI.† The Ir loading of Ir/SiO_2_ was calculated by ICP-AES to be 27.7 wt%. Fig. 1(b) shows XRD patterns for the catalysts. Both Ir/SiO_2_ and Ir black (AA) showed peaks ascribed to fcc Ir. A peak in the XRD pattern of Ir/SiO_2_ before 30° was ascribed to spherical silica particles, as shown in Fig. S1 in ESI.† The crystallite sizes were calculated using the Scherrer equation, which is expressed as *D* = *Kλ*/*β* cos *θ*, where Bragg angle is *θ*, shape factor is *K*, the full width half maximum is *β*, and the wave length of X-ray is *λ*. The crystallite size of Ir/SiO_2_ was calculated to be 1.7 nm from the highest peak near 40°, which is smaller than the crystallite size of 4.0 nm for Ir black (AA). Crystallite sizes of Ir/SiO_2_ synthesized using different concentrations of precursor were almost the same in this study, as shown in Fig. S5, S6, and Table S2 in ESI.†Fig. 1(c)–(g) show BF-STEM and SE-STEM images of Ir/SiO_2_. Ir nanoparticles were coated onto silica with high density and connected each other without aggregation. The size of the Ir nanoparticles was almost the same with an average diameter and standard deviation of 1.8 ± 0.3 nm, which was calculated from the diameters of 32 particles in Fig. 1(e). The average diameter obtained by STEM almost agrees with that calculated by XRD. Fig. S2† shows TEM images of Ir black (AA). The particles overlapped each other and had a size of 2–5 nm. Thus, the results from XRD and STEM showed that Ir/SiO_2_ particles were smaller than the size of the commercial Ir catalyst, suggesting a higher surface area for Ir/SiO_2_. High-resolution STEM (Fig. 1(f) and (g)) shows that lattice fringes were randomly directed and each of the Iridium nanoparticles composing the connected Ir catalysts were polycrystalline. EDX results (Fig. 1(h)–(k)) showed nanoparticles contained Ir. EELS (Fig. 1(h) and (i)) results showed a peak near 50 eV due to iridium oxide^26^ at all positions, which showed that a part of the nanoparticles were iridium oxide.

Electrochemical characterization of the catalysts was then performed using cyclic voltammetry (CV) to determine the electrochemical surface area (ECSA) and OER activity of Ir/SiO_2_ and Ir black (AA) on glassy carbon electrodes in 0.1 M HClO_4_ aq. A potential range of 0.05–1.5 V was used for the pretreatment,^14^ 0.4–1.4 V for ECSA and 1.2–1.8 V for OER. Fig. 2(a) and (b) shows cyclic voltammograms before and after pretreatment. Ir/SiO_2_ before the pretreatment showed peaks due to hydrogen adsorption and desorption at a potential of less than 0.3 V; these peaks did not appear after the pretreatment. Hydrogen adsorption and desorption occurs only on a metallic Ir surface and not on an IrO_2_ surface.^7,27^ Thus, the metallic Ir surface of Ir/SiO_2_ was oxidized to IrO_2_ by the pretreatment. On the other hand, the hydrogen adsorption and desorption peaks were not observed for the Ir black (AA) even before the pretreatment, and the cyclic voltammograms were observed to be almost the same before and after the pretreatment, which shows that the surface of Ir black (AA) was oxidized before the pretreatment. For confirmation of the relationship between hydrogen adsorption/desorption and the state of the iridium catalyst, the CVs and XPS was measured for the catalyst before and after heat treatment in air at 300 °C. The CV after heat treatment (Fig. S7†) showed that the peaks due to hydrogen adsorption and desorption did not appear as they did for Ir/SiO_2_ after the electrochemical pretreatment. XPS results showed that the as-synthesized Ir/SiO_2_ had 40% metallic iridium and 58% oxidized iridium, and almost all the metallic iridium was oxidized after heat treatment, as shown in Table S3.†

**Fig. 2:**
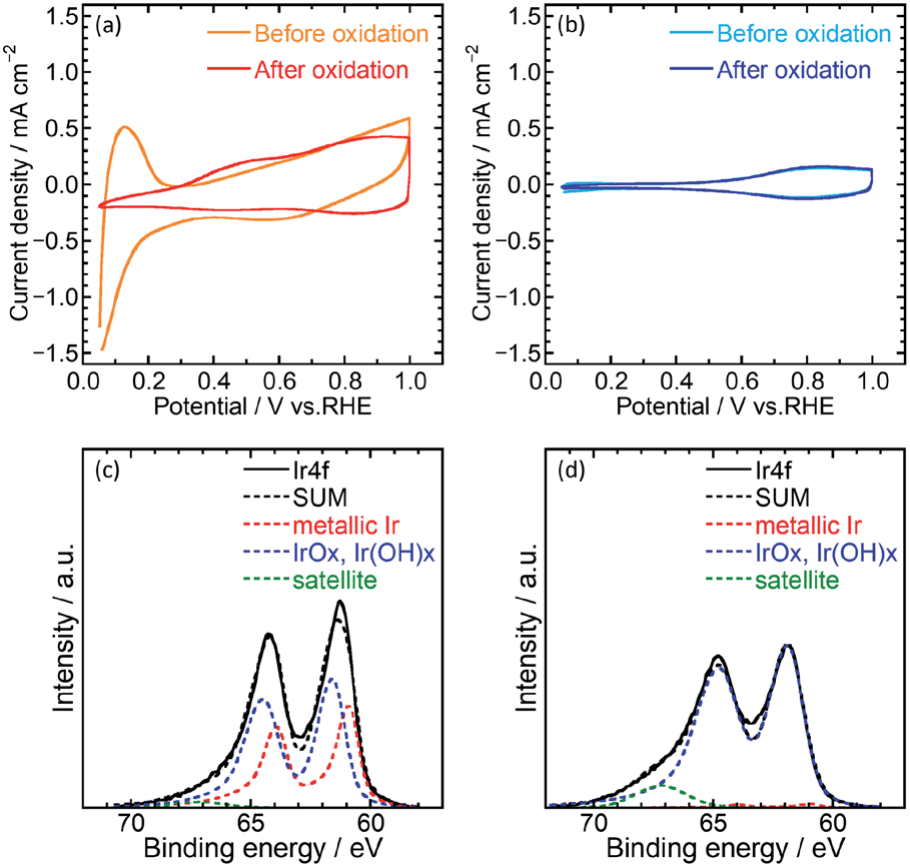
Cyclic voltammograms before and after oxidation of (a) Ir/SiO_2_ and (b) Ir black (AA). The voltammograms were measured in 0.1 M HClO_4_ aq. Ir 4f and curves of best fit from XPS analysis of Ir/SiO_2_ (c) before and (d) after heat treatment.

When the surface of the catalysts is composed of metallic Ir, ECSA can be usually calculated from the charge of the hydrogen desorption. However, because the surface of Ir black (AA) was composed of IrO_2_, ECSA was calculated from the capacitance of IrO_2_ after the pretreatment for both Ir/SiO_2_ and Ir black (AA). The capacitances were calculated from the average absolute current for anodic and cathodic sweeps, and was then converted to the ECSA with a specific capacitance 650 μF cm^−2^ for IrO_2_ (100).^11,28^ Fig. S3(a) and (b)† shows cyclic voltammograms with various potential sweep rates, from which the average anodic and cathodic currents were calculated from the currents at 0.65–0.75 V, as shown in Fig. S3(c).† The ECSA and standard deviation for Ir/SiO_2_ was determined to be 76.5 ± 1.9 m^2^ g^−1^, which is 2.6 times higher than that of Ir black (AA), 29.7 ± 1.5 m^2^ g^−1^. For the case of Ir/SiO_2_, the ECSA was also calculated from the charge of the hydrogen desorption before the pretreatment using a conversion factor of 179 μC cm_Ir_^−2^ for comparison.^27,29^ The ECSA and standard deviation was determined to be 67.6 ± 1.4 m^2^ g^−1^, which is almost the same as that obtained from the capacitance of IrO_2_. The reason for the high ECSA of Ir/SiO_2_ was ascribed to the formation of electron-conducting networks by small nanoparticles without aggregation.

The OER performance was then evaluated. CVs for the OER were measured at a scan rate of 10 mV s^−1^ and rotation speed of 1600 rpm. IR-free OER curves were obtained by averaging the current densities of anodic and cathodic scans, and then removing the overpotential due to an ohmic resistance calculated by multiplying the measured current (*I*) and cell resistance (*R*) using the cyclic voltammetry and the solution resistance measurement, respectively. Fig. 3(a) shows the OER curves for the catalysts. Ir/SiO_2_ showed a higher OER performance compared to Ir black (AA) with overpotentials of 305 mV and 374 mV, respectively. Mass activities were calculated from the currents at 1.48 V and the Ir loadings on the electrodes (9–10 μg cm^−2^). Ir/SiO_2_ showed a 5.2 times higher mass activity and 2.6 times larger ECSA, which means twice the surface area specific activity compared to Ir black (AA). Tafel slopes in the low current density region showed similar values of 33.8 mV dec^−1^ and 32.8 mV dec^−1^ for Ir/SiO_2_ and Ir black (AA), respectively, as shown in Fig. S4.† The rate determining steps for Ir/SiO_2_ and Ir black (AA) were considered to be similar because the Tafel slope depends on the rate determining step and is related to the bond strength of the catalyst surface and intermediates adsorbed onto the surface. The slope of 40 mV s^−1^ indicates that the rate determining step is the electron transfer reaction from S–OH to S–O, proton, and electron, where S denotes the surface, while the slope of 30 mV s^−1^ denotes that the rate determining step is a recombination reaction of OH and H from S–OH to S–O and water. In our case, either reaction or both reactions are the rate determining step because the two reactions have been reported to occur in parallel.^30^

**Fig. 3:**
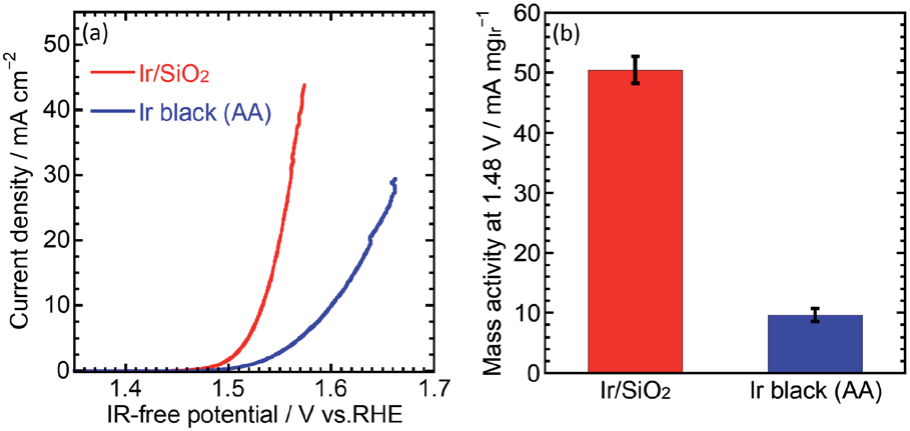
(a) OER curve for Ir/SiO_2_ and Ir black (AA) in 0.1 M HClO_4_ aq. at 1600 rpm. (b) Mass activity of Ir/SiO_2_ and Ir black calculated by dividing the current at 1.48 V by the mass of Ir on the electrode.

Then, the water electrolysis performance was evaluated with a membrane electrode assembly (MEA) fabricated using Ir/SiO_2_ as the anode catalyst, Nafion® membrane N115 (DuPont, thickness: 127 μm) as the electrolyte membrane, and platinum (Pt) supported on carbon (TKK, TEC10E50E) as the cathode catalyst. The Ir loading of the MEA was 0.3 mg cm^−2^, while the Pt loading was 0.3 mg cm^−2^. Water electrolysis and electrochemical impedance spectroscopy (EIS) were performed at 80 °C with circulating water in the anode flow channel. The resistance of a cell in the MEA was measured by EIS to be 191 mΩ cm^2^ at a cell potential of 1.5 V, which was similar to the value obtained for an MEA using N115, 197 mΩ cm^2^, in a previous report.^31^ These similar values showed that the catalyst layer using Ir/SiO_2_ had sufficient electron conductivity. Fig. 4 shows the result of the water electrolysis measurement. The MEA performance showed a potential of 1.76 V at 1.0 A cm^−2^ despite a low Ir loading of 0.3 mg cm^−2^. As shown in Table S2,† this value is better than, or at least comparable to, that of a few MEAs using Ir catalysts below the loading of 0.5 mg cm^−2^ and commercial Nafion® membranes with a thickness of more than 100 μm at the same temperature and atmospheric pressure, while general MEAs uses Ir loading of 1–4 mg cm^−2^. The MEA using connected Ir nanoparticle catalysts without any electron-conducting support showed sufficiently high performance. These results showed that Ir/SiO_2_ is promising for use as an anode catalyst for water electrolysis.

**Fig. 4:**
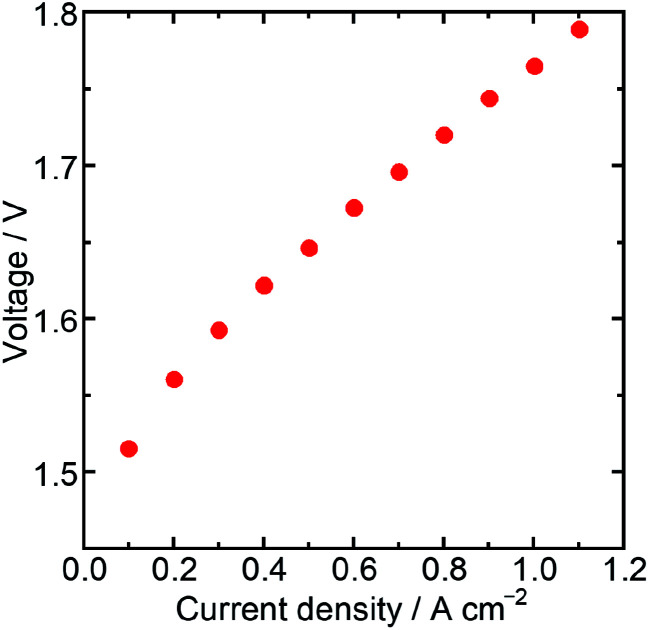
Water electrolysis performance of an MEA using Ir/SiO_2_ as an anode catalyst.

## Conclusions

We successfully synthesized connected Ir nanoparticle catalysts (Ir/SiO_2_) for OER without the need for an electron-conducting support such as carbon. Electron-conducting networks formed by a high density of Ir nanoparticles on silica prevent aggregation of the nanoparticles and eliminate the need for a carbon support, which enables one to operate at high potential. The particle size of Ir/SiO_2_ was calculated to be 1.8 nm by STEM; the ECSA of Ir/SiO_2_ was determined to be 2.6 times larger than that of Ir black (AA). The mass activity at 1.48 V was 5.2 times higher than that of the Ir black (AA) due to the high surface area of Ir/SiO_2_. An MEA using Ir/SiO_2_ as the anode catalyst showed sufficiently high performance despite a relatively low Ir loading of 0.3 mg cm^−2^. The results showed that Ir/SiO_2_ is promising for use as an anode catalyst for water electrolysis. The water electrolysis performance is expected to be enhanced by the optimization of the catalyst/electrode/MEA structure.

## Conflicts of interest

There are no conflicts to declare.

## Supplementary Material

NA-002-C9NA00568D-s001
