# Supplementary material for: Connected iridium nanoparticle catalysts coated onto silica with high density for oxygen evolution in polymer electrolyte water electrolysis
Source: Nanoscale Adv. 2019 Dec 2;2(1):171–5. doi: 10.1039/c9na00568d (PMC9419584; doi:10.1039/c9na00568d)
Supplement: NA-002-C9NA00568D-s001 [file NA-002-C9NA00568D-s001.pdf]

Electronic Supplementary Material (ESI) for Nanoscale Advances.
This journal is © The Royal Society of Chemistry 2019

Supplementary information

Connected iridium nanoparticle catalysts coated onto silica with high density for oxygen evolution in polymer electrolyte water electrolysis

Yoshiyuki Sugita, Takanori Tamaki, Hidenori Kuroki, Takeo Yamaguchi

Materials

Poly(diallyldimethylammonium chloride) solution (PDDA, 35 wt% in H_2O), tetraethylene glycol (TEG, 99%) and Nafion® perfluorinated resin solution (5 wt% Nafion® solution, 20 wt% Nafion® solution) were purchased from Sigma-Aldrich Co. LLC, United States. Tris(2,4-pentanedionato)iridium(Ⅲ) (Ir acac, Ir: 38.0–41.0%) was purchased from Tokyo Chemical Industry Co., Ltd., Japan. Ethanol (EtOH, 99.5%), 1-propanol (NPA, 99.5%), 2-propanol (IPA, 99.7%) and perchloric acid ($HClO_4$, 60.0–62.0%) were purchased from FUJIFILM Wako Pure Chemical Corporation, Japan. Iridium black (99.8%) was purchased from Alfa Aesar. United States. Platinum supported on carbon (Pt/C, Pt: 46.6%, TEC10E50E) was purchased from Tanaka Kikinzoku Kogyo Co., Ltd., Japan. Spherical silica particles (seahostar® KE-P30, diameter: 300 nm) was purchased from Nippon Shokubai Co., Ltd., Japan. Nafion® membrane (N-115, thickness: 127 μm) was purchased from DuPont, United States.

Synthesis of Ir/SiO_2

Connected Ir nanoparticle catalysts (Ir/SiO_2) were synthesized by 2 steps: modification of the SiO_2 surface by applying a coating of PDDA and the formation of Ir nanoparticles on PDDA/SiO_2 in a similar way as for the connected Pt-Fe nanoparticle catalysts.[1,2] Spherical silica particles (0.16 g) and ultra-pure water (5.0 mL, 18.2 MΩ cm, supplied by Direct-Q® 5 UV,

Merck Millipore, United States) were dispersed in a 50 mL centrifuge tube with ultrasonication for 1 hour. PDDA solution (12 g) and ultra-pure water (19.0 mL) were mixed in another 50 mL centrifuge tube with a vortex for 5 min; then, the SiO_2 dispersion was added to the PDDA solution. The mixture was dispersed with ultrasonication for 1 hour and then cleaned by centrifugation with ultra-pure water (25 mL). After 4 cycles of centrifugation, ultra-pure water (5 mL) was added to the precipitated silica; then, the mixture was dispersed with a vortex. The silica dispersion (approximately 4.5 mL, PDDA/SiO_2: 120 mg) and TEG (30 mL) were evaporated in a 100 mL eggplant flask at 80 °C for removing water; then, the mixture was cooled in a refrigerator. Ir acac (673.4 mg, 1.38 mol (9.2 mol/L (M))) and TEG (100 mL) were dispersed in a 500 mL three-neck flask with ultrasonication for several hours before adding the silica dispersion. After stirring at 50 °C for 16 hours, the mixture was bubbled with argon and hydrogen for 30 min and then heated to 230 °C for 2 hours with stirring to form Ir nanoparticles on the silica by polyol reaction. After cooling, the mixture was cleaned by 4 cycles of centrifugation with EtOH (25 mL). Then, Ir/SiO_2 powder was obtained by removing EtOH by evaporation. To oxidize Ir/SiO_2, the catalyst was heated at 300 °C for 1 hour in air. For comparison, Ir/SiO_2 was synthesized using different precursor concentrations of 8.1 M and 10.3 M, as shown in Fig. S5, S6, and Table S2. The obtained crystallite sizes were almost the same. On the other hand, with the lower precursor concentration of 8.1 M, silica surface could not be

covered completely (Fig. S5), and with the higher precursor concentration of 10.3 M, excess nanoparticles were formed on silica (Fig. S6). For control of nanoparticles sizes, Ir/SiO_2 was treated by the silica coating, the supercritical treatment, and the alkaline treatment. As shown in Table S2, crystallite sizes of the catalysts after the supercritical treatment at 330 °C or 380 °C were calculated to be 2.5 nm and 2.8 nm, respectively.

Structural characterization of the catalysts

The as-synthesized Ir/SiO_2 was characterized using inductively coupled plasma atomic emission spectroscopy (ICP-AES), X-ray diffraction (XRD), scanning transmission electron microscopy (STEM), energy dispersive X-ray spectrometry (EDX), electron energy loss spectroscopy (EELS), and X-ray photoelectron spectroscopy (XPS). For comparison, commercially available Ir black (Alfa Aesar (AA)) was used as a catalyst. The Ir loading for Ir/SiO_2 was measured by ICP-AES. Ir/SiO_2 (5 mg) was dissolved in aqua regia (10 mL) and hydrogen fluoride, which was diluted to 50 mL with water. Then, ICP-AES for the solution was measured using ICP Emission Spectroscopy ICPS-8100 (Shimadzu Corporation, Japan). XRD for the catalysts was conducted using a Multipurpose X-ray diffraction system Ultima IV (Rigaku Corporation, Japan) with a CuKα (λ = 0.154 nm) X-ray source. The peaks were ascribed with card data for fcc Ir (Inorganic Crystal Structure Database (ICSD) No. 64992). The powder X-ray analysis software PDXL 2.6

(Rigaku Corporation) was used for peak separation in the obtained XRD patterns, following which the Bragg angles and full width at half maximum (FWHM) were obtained. The crystallite sizes for the catalysts were calculated using the Scherrer equation[3], which is expressed as $D = K\lambda/\beta\cos\theta$, where the Bragg angle is θ, FWHM of the Ir (111) peak is β, wavelength of the X-ray is λ (0.154 nm) and the value of the shape factor is K (1.0747), assuming spherically shaped nanoparticles.[4] Field emission transmission electron microscope HF5000 (Hitachi High-Technologies Corporation, Japan) was used for STEM, EDX, and EELS analysis of Ir/SiO_2. Spherical aberration-corrected STEM/SEM HD-2700 (Hitachi High-Technologies) was used for parts of STEM of IrSiO_2, and a transmission electron microscope H-8100 (Hitachi High-Technologies) was used for transmission electron microscopy (TEM) of Ir/SiO_2 and Ir black (AA). The average diameter of Ir/SiO_2 obtained by STEM was calculated from the diameter values of 32 particles. XPS analysis was measured using PHI Quantera II (ULVAC-PHI, Inc., Japan).

Electrochemical measurements

Electrochemical characterization of the catalysts was then performed on glassy carbon electrodes (GCE). The Ir/SiO_2 catalysts ink was prepared by mixing Ir/SiO_2 (4.4 mg), 25 wt% IPA aq. (6.3 mL) and 5 wt% Nafion® solution (6.3 μL) with ultrasonication for 1 hour. Then, 10 μL of the

catalyst ink was drop cast onto a GCE, which was dried at room temperature with rotation at a speed of 600 rpm. A GCE for Ir black (AA) was prepared using almost the same method. The Ir loading for the Ir/SiO_2 and Ir black (AA) electrodes was 9.3 μg/cm^2 and 10.0 μg/cm^2, respectively. All electrochemical characterization of the catalysts was performed in 0.1 M $HClO_4$ aq. at room temperature with an electrochemical measurement system HZ-7000 (HOKUTO DENKO CORPORATION, Japan) using a reversible hydrogen electrode (RHE) as reference electrode and platinum electrode as counter electrode. The electrochemical surface area (ECSA) and OER activity of the catalysts were evaluated using cyclic voltammetry (CV) and IR measurement. A potential range of 0.05–1.5 V was used for the pretreatment,[5] 0.05–1.0 V for ECSA by the hydrogen desorption of metallic Ir, 0.4–1.4 V for ECSA by the capacitance of IrO_2 and 1.2–1.8 V for OER. Pretreatment was performed to oxidize the surfaces of the catalysts by CV of 50 cycles at a scan rate of 500 mV s^{-1}.[5] The ECSA was calculated from the capacitance of IrO_2 after the pretreatment of both Ir/SiO_2 and Ir black (AA). The capacitances were calculated from the average values of the absolute current for the anodic and cathodic sweeps; these values were then converted to an ECSA using the specific capacitance of IrO_2 (100), 650 of μF cm^{-2}.[6,7] Fig. S2(a) and (b) shows cyclic voltammograms with various potential sweep rates, from which the average anodic and cathodic currents were calculated using the currents at 0.65–0.75 V, as shown in Fig. S2(c). For comparison, the ECSA of Ir/SiO_2 was calculated using another method

based on the charge of the hydrogen desorption before the pretreatment with a conversion factor of 179 μC cm_{Ir}^{-2}.[8,9] The charge was calculated from the anodic current of the peak near 0.1 V after correcting for double layer charging by subtraction of the current at 0.3 V from the total current. CVs for OER were measured at a scan rate of 10 mV s^{-1} and rotation speed of 1600 rpm for 10 cycles. IR-free OER curves were obtained by averaging the current densities of the anodic and cathodic scans, and then removing the overpotential due to an ohmic resistance, which is dependent on the concentration of electrolyte, the distance between electrodes, and so forth. The overpotential was calculated by multiplying the measured current (I) and cell resistance (R) using the cyclic voltammetry and the solution resistance measurement, respectively. Subsequently, the IR-free curves were obtained by subtracting the overpotential from the measured potential, as shown in following literature. Mass activities for the OER were calculated using the IR-free currents at 1.48 V and the Ir loading on the electrode (9–10 μg cm^{-2}). The OER performance was evaluated based on the highest performance in 2–10 cycles for each catalyst: 2nd cycle for Ir/SiO_2, 5th cycle for Ir black (AA). The Tafel slope for the catalysts was calculated using Tafel plots for the IR-free OER curves in the low current density region below 1 mA cm^{-2}.

MEA measurements

A 3.5 cm square Nafion® membrane N115 was treated in 1 M HNO_3 aq. at 90 °C for 2 hours;

then, the membrane was cleaned in RO water (12.8 MΩ cm, supplied by Elix 3UV Essential, Merck Millipore) at 100 °C for 1 hour. The membrane electrode assembly (MEA) was made by using the treated Nafion® N115 as electrolyte membrane, Ir/SiO_2 as anode catalyst, Pt/C as cathode catalyst and Nafion® solution as ionomer. An ink of the anode catalyst was prepared by mixing Ir/SiO_2 (60 mg), 20 wt% Nafion® solution (11.25 mg), IPA (6.6 mL) and RO water (8.4 mL) with ultrasonication for a few hours, followed by treatment in an autoclave at 200 °C for 20 hours to ensure the formation of a uniform ionomer coating on the catalyst. After ultrasonication for 1 hour, the obtained ink was coated onto the membrane with a catalyst layer size of 5 cm^2 with a spray method using a pulse spray system (Nordson Corporation, United States). An ink of the cathode catalyst was prepared by mixing Pt/C (2 g), RO water (8.5 g), 20 wt% Nafion® solution (5.3 g), NPA (8.5 g) and IPA (8.5 g) with ball milling at a rotating rate of 200 rpm for 1 hour using zirconia balls with a diameter of 5 mm. A cathode catalyst layer was prepared using a decal method using the ink, which was then attached onto the opposite side of the membrane by pressing at 5.11 kN at 135 °C for 5 min to obtain an MEA. An electrolyzer cell (FC Development Co., Ltd., Japan) was set up with the MEA using carbon paper (SGL carbon, Germany) as a porous transfer layer. Electrochemical impedance spectroscopy (EIS) was measured using an electrochemical interface SI 1287 and impedance/gain-phase analyzer SI 1260 (TOYO Corporation, Japan) at a cell potential of 1.5 V in the frequency range of 1 Hz to

100 kHz. Water electrolysis was measured using battery charge/discharge system HJ1010SD8 (HOKUTO DENKO CORPORATION) from 0.1 A cm^{-2} to 1.1 A cm^{-2} with each current density value held constant for a few minutes. The EIS and water electrolysis performance of the cell was measured at 80 °C with water circulating in the anode flow channel at a flow rate of 10 mL min^{-1}.

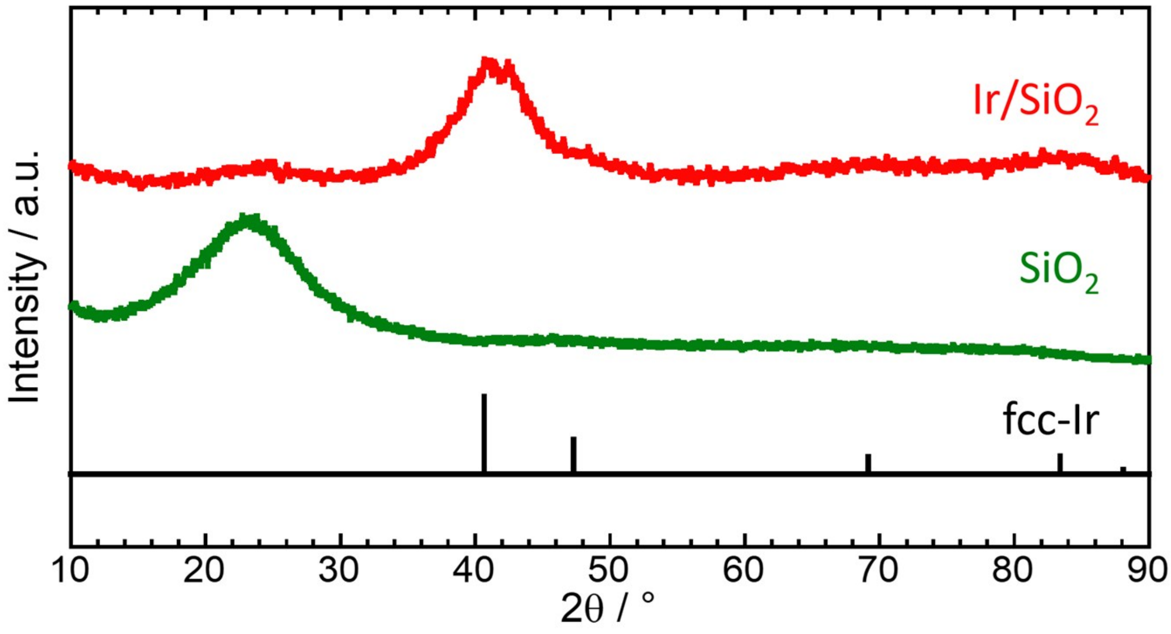

Fig. S1. XRD patterns for Ir/SiO_2 and spherical SiO_2 particles. The peak data for fcc-Ir was obtained from a database (ICSD No. 64992).

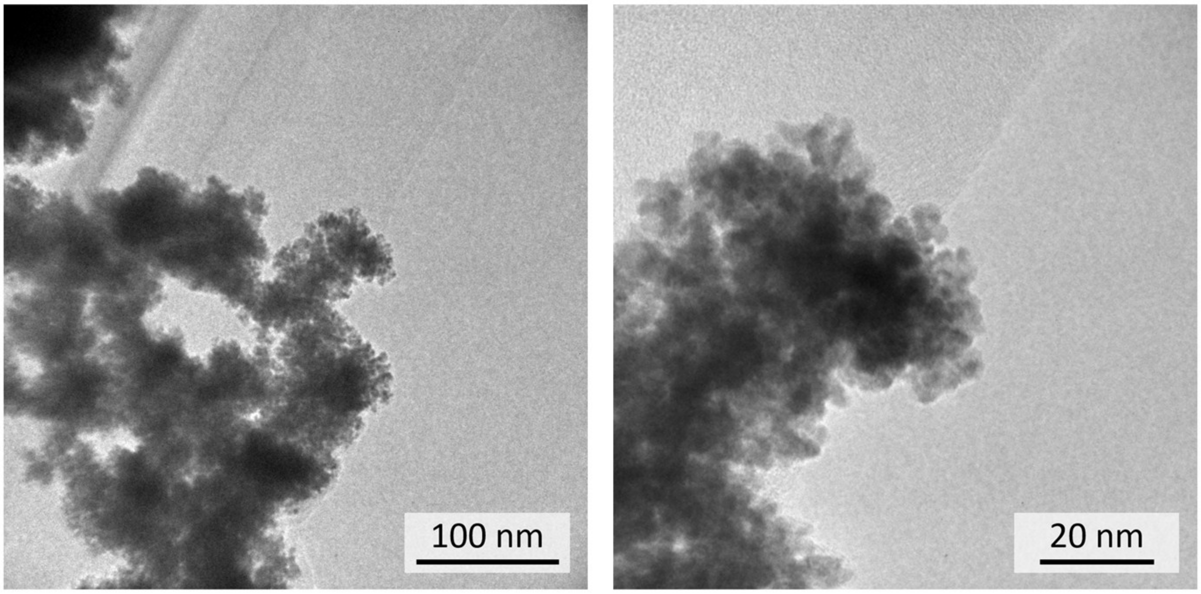

Fig. S2. TEM images of Ir black (AA).

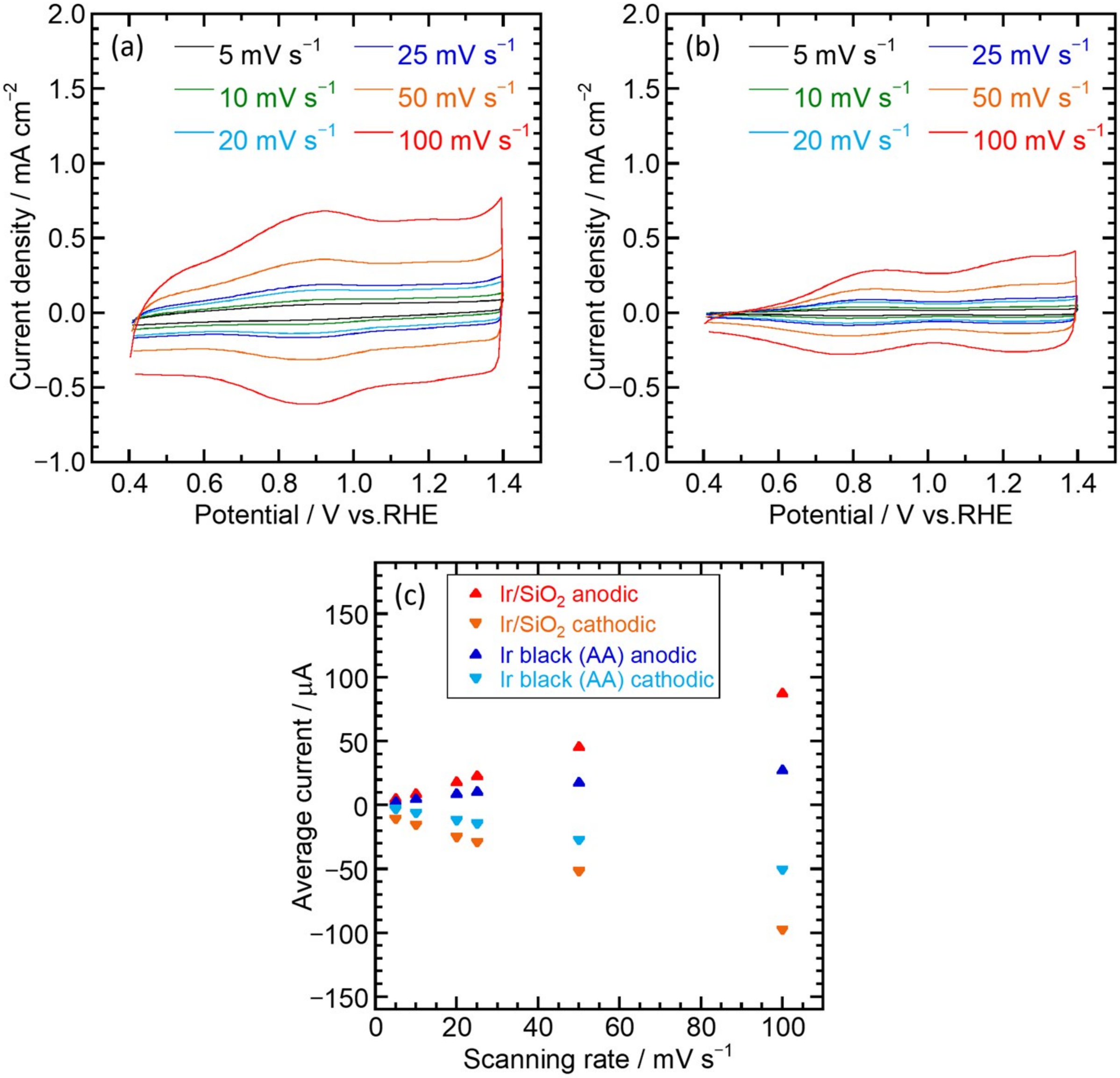

Fig. S3. Cyclic voltammograms for a varying scanning rate for (a) Ir/SiO_2 and (b) Ir black (AA). (c) Average current calculated from the currents measured at 0.65–0.75 V in (a) and (b).

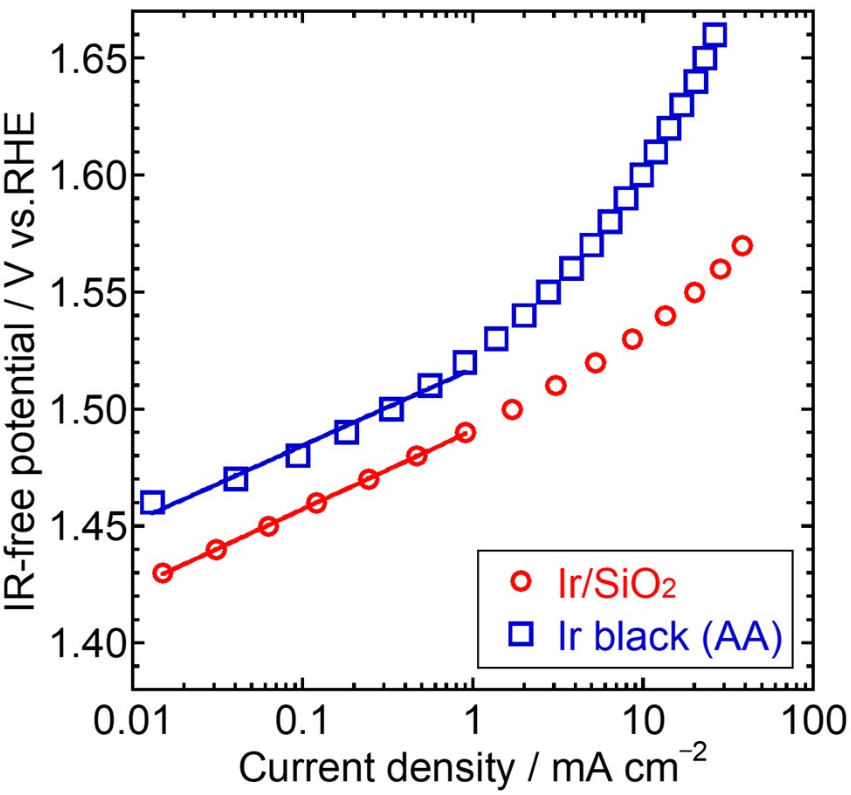

Fig. S4. Tafel plots from the OER curves of Ir/SiO_2 and Ir black (AA).

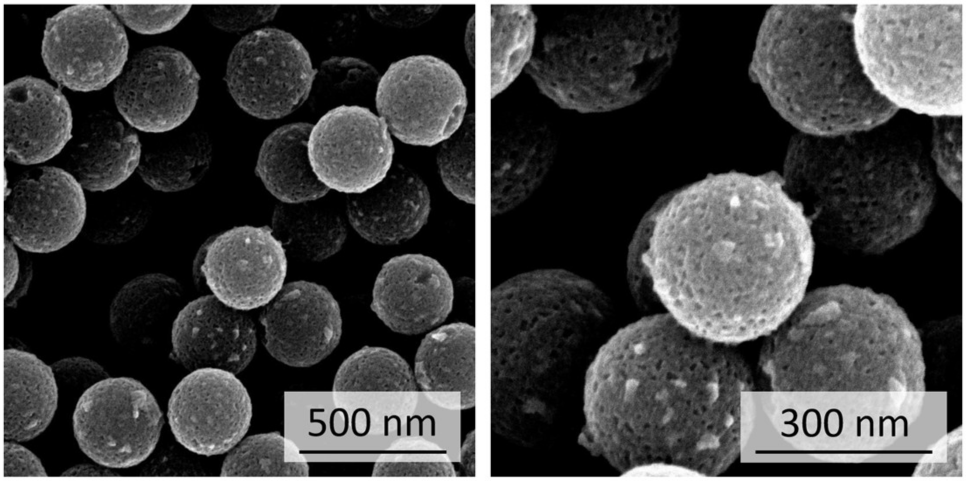

Fig. S5. SEM images of Ir/SiO_2 synthesized using the lower precursor concentration of 8.1 M.

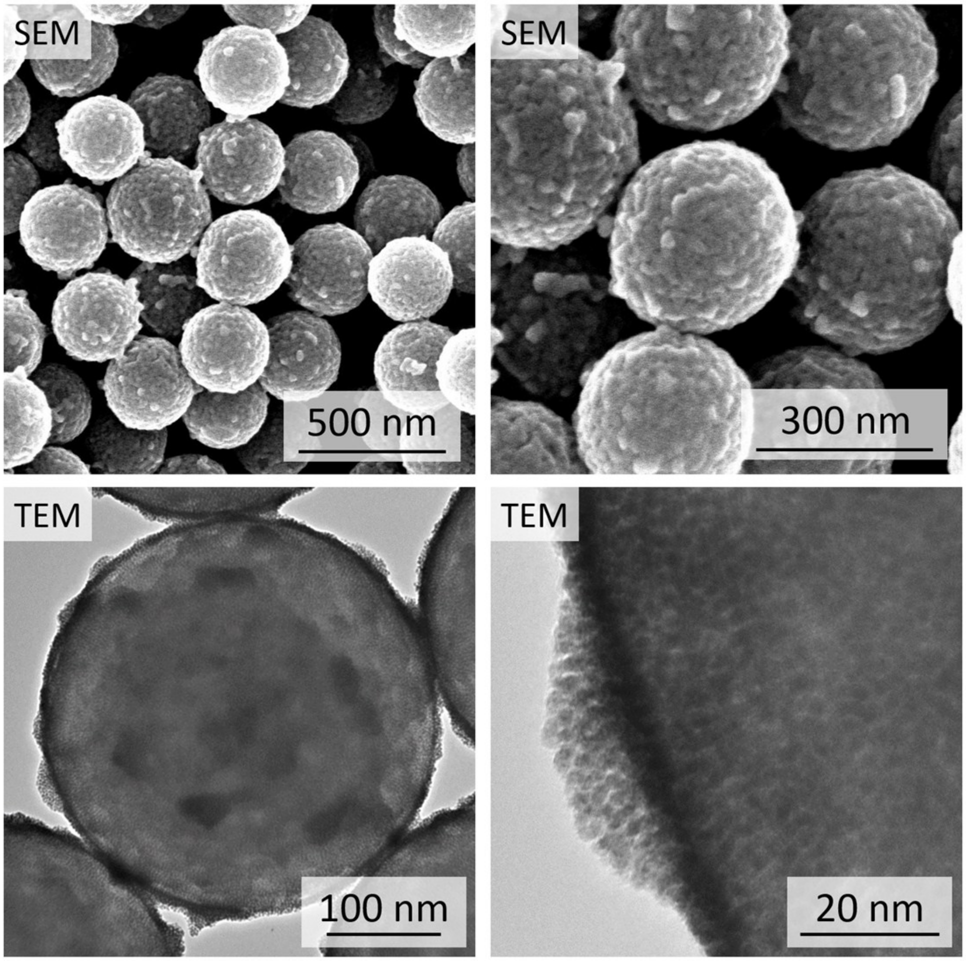

Fig. S6. SEM and TEM images of Ir/SiO_2 synthesized using the higher precursor concentration of 10.3 M.

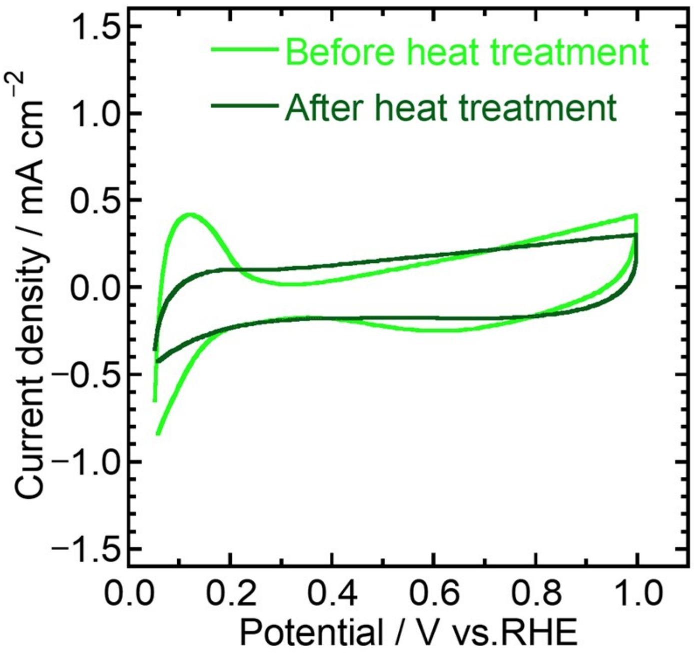

Fig. S7. Cyclic voltammograms of Ir/SiO_2 before and after heat treatment.

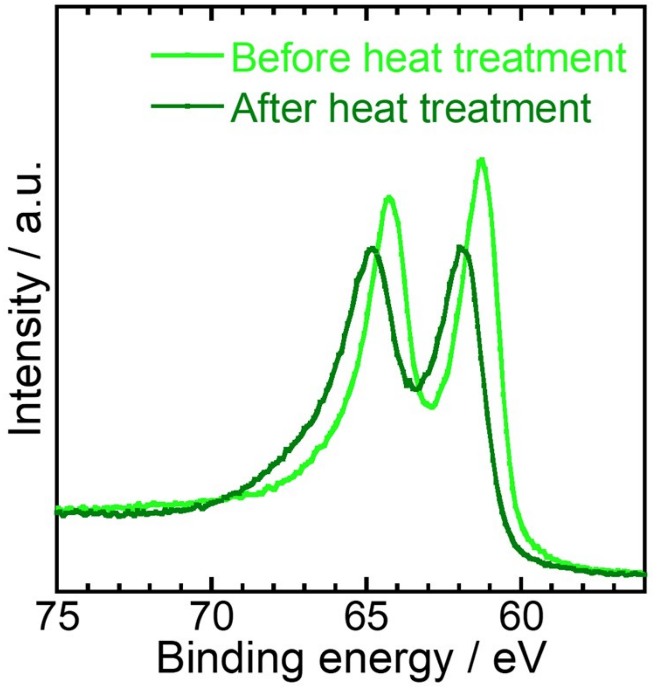

Fig. S8. Ir4f curves from XPS analysis of Ir/SiO_2 before and after heat treatment.

Table S1. Parameters and performance of Ir/SiO_2 and Ir black (AA).

catalyst	crystallite size by XRD [nm]	particle size by STEM/TEM [nm]	ECSA [m^2 g^{-1}]	overpotential @10 mA cm^{-2} [mV]	mass activity @1.48 V [mA mg^{-1}]
Ir/SiO_2	1.7	1.8 ± 0.3	76.5 ± 1.9	305 ± 2	50.5 ± 2.2
Ir black (AA)	4.0	(2–5)	29.7 ± 1.5	374 ± 3	9.6 ± 1.1

Table S2. Crystallite sizes of Ir/SiO_2 synthesized by each precursor conditions and after treatments such as silica coating, supercritical treatment (SCT) and alkaline treatment.

before or after treatments	crystallite size by XRD [nm]	amount of precursor [mol]	SCT temperature [°C]
Before (this study)	1.67 ± 0.06	1.38	-
Before	1.59 ± 0.04	1.21	-
Before	1.69 ± 0.02	1.54	-
After	2.5	1.21	330
After	2.8	1.21	380

Table S3. Composition of each iridium state of Ir/SiO_2 calculated from XPS analysis.

before or after heat treatment	metallic Ir [%]	IrOx, Ir(OH)x [%]	satellite [%]
Before	39.9	58.1	2.1
After	1.4	90.7	7.9

Table S4. Water electrolysis performance of MEAs using Ir-based catalysts as anode and Pt-based catalysts as cathode.

Ref.	Anode catalyst	Cathode catalyst	An. Ir loading [mg cm^{-2}]	Ca. Pt loading [mg cm^{-2}]	Electrolyte membrane	Pres -sure	Cell temp. [C °]	Cell voltage [V@1 A cm^{-2}]	CL. fabrication (An. / Ca.)
This Work	Ir/SiO_2	46%Pt/C (TKK)	0.3	0.3	N115	-	80	1.76	S-mem / Decal
10	Ir-black	40%Pt/C	1.4	0.5	N117	-	80	1.95	Decal
10	IrO_2 (AA)	40%Pt/C	1.1	0.2	N117	-	80	1.93	Decal
10	IrO_2 (AA)	40%Pt/C	1.4	0.2	N117	-	80	1.90	Decal
10	IrO_2 (AA)	40%Pt/C	1.7	0.2	N117	-	80	1.88	Decal
11	IrO_x/Nafion®	Pt/Vulcan	0.08	0.3	N117	2.76 MPa	80	1.88 @1.8 A cm^{-2}	RSDT
12	Ir-Ni nanowires	-	0.1	-	-	-	80	1.61	S (3M)
13	$Ir_{0.7}Ru_{0.3}O_x$	Pt/Vulcan	0.34 (noble)	0.1	Aquivion® (90 μm)	-	80	1.63	S-CC / Sp-CC
14	Ir-black/Ti_4O_7	Pt/C (JM)	0.42	0.5	N212	-	40	0.26 A mg^{-1} @1.7 V	SP
15	IrO_2 (1:100)-450 °C	Pt black	2.0	2.0	N115	-	80	1.649	S-mem / Sp-CC
16	IrO_2	46%Pt/C (TKK)	1.5	0.5	N117	0.1 MPa	80	1.66	S-mem
16	IrO_2	46%Pt/C (TKK)	1.5	0.5	N117	0.3 MPa	120	1.56	S-mem
17	IrO_2	30%Pt/C (Premetek)	4 (oxide)	1.8	N117	-	80	1.82	Decal/ S-CP
18	$Ir_{0.7}Ru_{0.3}O_x$ (EC)	40%Pt/C (JM)	1 (oxide)	0.4	N212	-	80	1.70	S-mem
19	IrO_2 (SK)	20%Pt/C (SH)	2 (oxide)	0.2	N117	-	80	1.72	S-mem-wet

AA = Alfa Aesar, EC = electrochemically leaching, JM = Johnson Matthey, RSDT = reactive spray deposition technology, S-CC/CP/mem = spray coating on carbon cloth/carbon paper/membrane, SH = Shanghai Hesen, SK = Shanxi Kaida, S-mem-wet = spray coating on swelling membrane with hot water, SP = screen printing, Sp-CC = spread on carbon cloth, TKK= Tanaka Kikinzoku Kogyo.

Table S4. (continued)

Ref.	Anode catalyst	Cathode catalyst	An. Ir loading [mg cm^{-2}]	Ca. Pt loading [mg cm^{-2}]	Electrolyte membrane	Pres-sure	Cell temp. [C °]	Cell voltage [V@1 A cm^{-2}]	CL. fabrication (An. / Ca.)
20	40Ir/TV-20	40%Pt/C (JM)	2.5	0.5	N117	-	80	2.02	S-mem
21	IrO_2 (SC)	46%Pt/C (TKK)	0.1	0.25	N115	-	80	1.89	S-Decal
21	IrO_2 (SC)	46%Pt/C (TKK)	0.3	0.25	N115	-	80	1.79	S-Decal
21	IrO_2 (SC)	46%Pt/C (TKK)	0.7	0.25	N115	-	80	1.71	S-Decal
21	IrO_2 (SC)	46%Pt/C (TKK)	1.0	0.25	N115	-	80	1.73	S-Decal
21	IrO_2 (SC)	46%Pt/C (TKK)	1.2	0.25	N115	-	80	1.73	S-Decal
21	IrO_2 (SC)	46%Pt/C (TKK)	1.7	0.25	N115	-	80	1.71	S-Decal
22	IrO_2/Ti	46%Pt/C (TKK)	0.12	0.25	N115	-	80	1.73	S-Decal
23	IrO_2 (AA)	46%Pt/C (TKK)	2	0.4	N212	2.5 bar	120	1.50 @0.91 A cm^{-2}	S-CP
23	IrO_2/CP	46%Pt/C (TKK)	0.1	0.4	N212	2.5 bar	120	1.50 @0.76 A cm^{-2}	ED / S-CP
24	IrO_2/TiO_2 (Umicore)	46%Pt/C (TKK)	2.0	0.35	N212	1 bar	80	1.59	Decal
25	Ir black (JM)	Pt/C (TKK)	0.1	0.4	N1110	-	80	1.77	Decal
25	Ir black (JM)	Pt/C (TKK)	0.4	0.4	N1110	-	80	1.72	Decal
26	IrO_2/CP	46%Pt/C (TKK)	0.1	0.4	N212	-	90	1.60	ED / S-CP
27	$Ir_{0.7}Ru_{0.3}O_x$	Pt/Vulcan (E-TEK)	1.5 (oxide)	0.5	N115	-	80	1.60	S-mem / Sp-CC
27	IrO_x	Pt/Vulcan (E-TEK)	1.5 (oxide)	0.5	N115	-	80	1.65	S-mem / Sp-CC
28	60%IrO_2/ATO	20%Pt/C (AA)	1	0.5	N115	-	80	1.80	S-mem
29	90%IrO_2/ITO	20%Pt/C (AA)	2 (oxide)	0.5	N115	-	80	1.73	S-mem

AA = Alfa Aesar, ATO = antimony doped tin oxide, CP = carbon paper, ED = electrodeposition, ITO = indium tin oxide, JM = Johnson Matthey, SC = Surepure® Chemetals, S-CP/mem = spray coating on carbon paper/membrane, S-Decal = spray coating on PTFE and

then decal method, SP = screen printing, Sp-CC = spread on carbon cloth, TKK= Tanaka Kikinzoku Kogyo.

Table S4. (continued)

Ref.	Anode catalyst	Cathode catalyst	An. Ir loading [mg cm^{-2}]	Ca. Pt loading [mg cm^{-2}]	Electrolyte membrane	Pres -sure	Cell temp. [C °]	Cell voltage [V@1 A cm^{-2}]	CL. fabrication (An. / Ca.)
5	Ir-ND/ATO	46%Pt/C (TKK)	1.0	0.4	N212	-	80	1.67	S-mem
5	Ir-ND/Carbon	46%Pt/C (TKK)	1.0	0.4	N212	-	80	1.68	S-mem
5	Ir-ND	46%Pt/C (TKK)	1.0	0.4	N212	-	80	1.71	S-mem
30	$Ir_{0.6}Sn_{0.4}O_2$	70%Pt/C	1.5 (oxide)	0.4	N115	-	80	1.63	S-mem
31	$Ru_{0.9}Ir_{0.1}O_2$	46%Pt/C (TKK)	1.0 (oxide)	0.18	N117	-	80	1.68	S-Decal / Decal
32	$Ru_{0.7}Ir_{0.3}O_2$	46%Pt/C (TKK)	2.5 (oxide)	0.4	N115	-	r.t.	2.30	Decal
33	IrO_2/SnO_2 (2:1)	40%Pt/C (JM)	1	0.2	N212	-	80	1.60	S-Decal
34	$Ru_{0.3}Ir_{0.7}O_2$/ $Pt_{0.15}$	40%Pt/Vulcan (JM)	1.8	0.3	N117	-	60	1.76	S-mem
35	IrO_2 (JM)	40%Pt/C (JM)	0.5	0.5	N212	-	80	1.67	S-mem-IR
35	IrO_2 (JM)	40%Pt/C (JM)	1	0.5	N212	-	80	1.62	S-mem-IR
35	IrO_2 (JM)	40%Pt/C (JM)	1.5	0.5	N212	-	80	1.58	S-mem-IR
35	IrO_2 (JM)	40%Pt/C (JM)	2	0.5	N212	-	80	1.57	S-mem-IR
36	Ir black	Pt/Vulcan	2	0.8	N115	-	90	1.72	-
36	Ir black	Pt/GNF	2	0.8	N115	-	90	1.67	-
37	IrO_2/SnO_2	40%Pt/C (JM)	1.5 (oxide)	0.5 (Pt/C)	N212	-	80	1.58	S-Decal
37	IrO_2/SnO_2	40%Pt/C (JM)	1.5 (oxide)	0.5 (Pt/C)	N212	-	80	1.60	S-Decal
38	Ir black	40%Pt/Vulcan	2	0.7	N115	1 bar	90	1.67	S-Ti

ATO = antimony doped tin oxide, ED = electrodeposition, GNF = graphitic nano-fibers, JM = Johnson Matthey, ND = nanodendrites, r.t. = room temperature, S-Decal = spray coating on PTFE and then decal method, S-mem/Ti = spray coating on membrane/porous-Ti,

S-mem-IR = spray coating on membrane with infrared radiation, TKK= Tanaka Kikinzoku Kogyo.

Table S4. (continued)

Ref.	Anode catalyst	Cathode catalyst	An. Ir loading [mg cm^{-2}]	Ca. Pt loading [mg cm^{-2}]	Electrolyte membrane	Pres -sure	Cell temp. [C °]	Cell voltage [V@1 A cm^{-2}]	CL. fabrication (An. / Ca.)
39	$Ru_{0.7}Ir_{0.3}O_2$	20%Pt/C (AA)	1.5 (oxide)	0.5 (Pt/C)	N117	-	80	1.586	S-Decal
40	IrO_2	28%Pt/C (TKK)	1.5 (oxide)	0.5 (Pt/C)	N1035	-	80	1.67	S-mem
40	IrO_2	28%Pt/C (TKK)	1.5 (oxide)	0.5 (Pt/C)	N1035	-	80	1.60	S-mem
41	$Ir_{0.4}Ru_{0.6}O_2$	28%Pt/C (TKK)	1.5 (oxide)	0.5 (Pt/C)	N1035	-	80	1.646	S-mem
41	$Ir_{0.4}Ru_{0.6}Mo_xO_y$	28%Pt/C (TKK)	1.5 (oxide)	0.5 (Pt/C)	N1035	-	80	1.606	S-mem
42	Ir black	Pt/C	2.0	0.8 (Pt/C)	N115	1 bar	90	1.68	S-mem
43	Ir black	Pt/Vulcan	2.4	0.7	N115	-	90	1.68	S-Ti
44	Ir	28%Pt/C (TKK)	3 (oxide)	0.5 (Pt/C)	N112	-	80	1.72	S-mem
44	IrO_2 (A)	28%Pt/C (TKK)	3 (oxide)	0.5 (Pt/C)	N112	-	80	1.66	S-mem
44	IrO_2 (C)	28%Pt/C (TKK)	3 (oxide)	0.5 (Pt/C)	N112	-	80	1.62	S-mem
44	$Ru_{0.5}Ir_{0.5}O_2$	28%Pt/C (TKK)	3 (oxide)	0.5 (Pt/C)	N112	-	80	1.65	S-mem
45	IrO_2	Pt black (SCR)	2.5	2	N115	-	80	1.60	S-mem
46	IrO_2	Pt/Vulcan (E-TEK)	2 (oxide)	0.4	N115	-	80	1.65	S-mem

AA = Alfa Aesar, IrO_2 (A) = IrO_2 prepared by Adams method, IrO_2 (C) = IrO_2 prepared by colloid method, JM = Johnson Matthey, ND = nanodendrites, SCR = Shanghai Chemical Reagent, S-Decal = spray coating on PTFE and then decal method, S-mem/Ti = spray coating on membrane/porous-Ti, S-mem-IR = spray coating on membrane with infrared radiation, TKK= Tanaka Kikinzoku Kogyo.

References

1. H. Kuroki, T. Tamaki, M. Matsumoto, M. Arao, Y. Takahashi, H. Imai, Y. Kitamoto and T. Yamaguchi, *ACS Appl. Energy Mater.*, 2018, **1**, 324–330.
2. T. Tamaki, H. Kuroki, S. Ogura, T. Fuchigami, Y. Kitamoto and T. Yamaguchi, *Energy Environ. Sci.*, 2015, **8**, 3545–3549.
3. P. Scherrer, in *Nachrichten von der Gesellschaft der Wissenschaften zu Göttingen: Philologisch-Historische Klasse*, Commissionsverlag der Dieterich'schen Verlagsbuchhandlung, Gottingen, 1918, pp. 98–100.
4. J. I. Langford and A. J. C. Wilson, *J. Appl. Crystallogr.*, 1978, **11**, 102–113.
5. H. S. Oh, H. N. Nong, T. Reier, M. Gliech and P. Strasser, *Chem. Sci.*, 2015, **6**, 3321–3328.
6. B. M. Tackett, W. Sheng, S. Kattel, S. Yao, B. Yan, K. A. Kuttiyiel, Q. Wu and J. G. Chen, *ACS Catal.*, 2018, **8**, 2615–2621.
7. T. Hepel, F. H. Pollak and W. E. O'Grady, *J. Electrochem. Soc.*, 1985, **132**, 2385–2390.
8. S. M. Alia, K. E. Hurst, S. S. Kocha and B. S. Pivovar, *J. Electrochem. Soc.*, 2016, **163**, F3051–F3056.
9. R. Woods, *J. Electroanal. Chem. Interfacial Electrochem.*, 1974, **49**, 217–226.

10. H. J. Ban, M. Y. Kim, D. Kim, J. Lim, T. W. Kim, C. Jeong, Y. A. Kim and H. S. Kim, *J. Electrochem. Sci. Technol.*, 2019, **10**, 22–28.

11. H. Yu, N. Danilovic, Y. Wang, W. Willis, A. Poozhikunnath, L. Bonville, C. Capuano, K. Ayers and R. Maric, *Appl. Catal. B Environ.*, 2018, **239**, 133–146.

12. S. M. Alia, S. Shulda, C. Ngo, S. Pylypenko and B. S. Pivovar, *ACS Catal.*, 2018, **8**, 2111–2120.

13. S. Siracusano, V. Baglio, N. Van Dijk, L. Merlo and A. S. Aricò, *Appl. Energy*, 2017, **192**, 477–489.

14. T. Lagarteira, F. Han, T. Morawietz, R. Hiesgen, D. Garcia Sanchez, A. Mendes, A. Gago and R. Costa, *Int. J. Hydrogen Energy*, 2018, **43**, 16824–16833.

15. G. Li, S. Li, M. Xiao, J. Ge, C. Liu and W. Xing, *Nanoscale*, 2017, **9**, 9291–9298.

16. H. Li, H. Nakajima, A. Inada and K. Ito, *Int. J. Hydrogen Energy*, 2018, **43**, 8600–8610.

17. L. Jinkyu, P. Dongmin, J. S. Seo, R. Chi-Woo, C. Juhyuk, Y. Daejin, P. Minju, J. Hyeyoung and L. Hyunjoo, *Adv. Funct. Mater.*, 2018, **28**, 1704796.

18. L. Wang, V. A. Saveleva, S. Zafeiratos, E. R. Savinova, P. Lettenmeier, P. Gazdzicki, A. S. Gago and K. A. Friedrich, *Nano Energy*, 2017, **34**, 385–391.

19. Y. Shi, Z. Lu, L. Guo and C. Yan, *Int. J. Hydrogen Energy*, 2017, **42**, 26183–26191.

20. C. Hao, H. Lv, Q. Zhao, B. Li, C. Zhang, C. Mi, Y. Song and J. Ma, *Int. J. Hydrogen Energy*, 2017, **42**, 9384–9395.

21. C. Rozain, E. Mayousse, N. Guillet and P. Millet, *Appl. Catal. B Environ.*, 2016, **182**, 153–160.

22. C. Rozain, E. Mayousse, N. Guillet and P. Millet, *Appl. Catal. B Environ.*, 2016, **182**, 123–131.

23. B. S. Lee, H. Y. Park, I. Choi, M. K. Cho, H. J. Kim, S. J. Yoo, D. Henkensmeier, J. Y. Kim, S. W. Nam, S. Park, K. Y. Lee and J. H. Jang, *J. Power Sources*, 2016, **309**, 127–134.

24. M. Bernt and H. A. Gasteiger, *J. Electrochem. Soc.*, 2016, **163**, F3179–F3189.

25. S. M. Alia, B. Rasimick, C. Ngo, K. C. Neyerlin, S. S. Kocha, S. Pylypenko, H. Xu and B. S. Pivovar, *J. Electrochem. Soc.*, 2016, **163**, F3105–F3112.

26. B. S. Lee, S. H. Ahn, H. Y. Park, I. Choi, S. J. Yoo, H. J. Kim, D. Henkensmeier, J. Y. Kim, S. Park, S. W. Nam, K. Y. Lee and J. H. Jang, *Appl. Catal. B Environ.*, 2015, **179**, 285–291.

27. S. Siracusano, N. Van Dijk, E. Payne-Johnson, V. Baglio and A. S. Aricò, *Appl. Catal. B Environ.*, 2015, **164**, 488–495.

28. V. K. Puthiyapura, M. Mamlouk, S. Pasupathi, B. G. Pollet and K. Scott, *J. Power Sources*, 2014, **269**, 451–460.

29. V. K. Puthiyapura, S. Pasupathi, H. Su, X. Liu, B. Pollet and K. Scott, *Int. J. Hydrogen Energy*, 2014, **39**, 1905–1913.

30. G. Li, H. Yu, X. Wang, D. Yang, Y. Li, Z. Shao and B. Yi, *J. Power Sources*, 2014, **249**, 175–184.

31. T. Audichon, E. Mayousse, S. Morisset, C. Morais, C. Comminges, T. W. Napporn and K. B. Kokoh, *Int. J. Hydrogen Energy*, 2014, **39**, 16785–16796.

32. N. Mamaca, E. Mayousse, S. Arrii-Clacens, T. W. Napporn, K. Servat, N. Guillet and K. B. Kokoh, *Appl. Catal. B Environ.*, 2012, **111**, 376–380.

33. J. Xu, G. Liu, J. Li and X. Wang, *Electrochim. Acta*, 2012, **59**, 105–112.

34. C. Xu, L. Ma, J. Li, W. Zhao and Z. Gan, *Int. J. Hydrogen Energy*, 2012, **37**, 2985–2992.

35. H. Su, B. J. Bladergroen, V. Linkov, S. Pasupathi and S. Ji, *Int. J. Hydrogen Energy*, 2011, **36**, 15081–15088.

36. S. A. Grigoriev, M. S. Mamat, K. A. Dzhus, G. S. Walker and P. Millet, *Int. J. Hydrogen Energy*, 2011, **36**, 4143–4147.

37. J. Xu, R. Miao, T. Zhao, J. Wu and X. Wang, *Electrochem. Commun.*, 2011, **13**, 437–439.

38. P. Millet, R. Ngameni, S. A. Grigoriev, N. Mbemba, F. Brisset, A. Ranjbari and C. Etiévant, *Int. J. Hydrogen Energy*, 2010, **35**, 5043–5052.

39. W. Xu and K. Scott, *Int. J. Hydrogen Energy*, 2010, **35**, 12029–12037.

40. J. Cheng, H. Zhang, G. Chen and Y. Zhang, *Electrochim. Acta*, 2009, **54**, 6250–6256.

41. J. Cheng, H. Zhang, H. Ma, H. Zhong and Y. Zou, *Int. J. Hydrogen Energy*, 2009, **34**, 6609–6613.

42. S. A. Grigoriev, P. Millet, S. A. Volobuev and V. N. Fateev, *Int. J. Hydrogen Energy*, 2009, **34**, 4968–4973.

43. S. A. Grigoriev, P. Millet and V. N. Fateev, *J. Power Sources*, 2008, **177**, 281–285.

44. S. Song, H. Zhang, X. Ma, Z. Shao, R. T. Baker and B. Yi, *Int. J. Hydrogen Energy*, 2008, **33**, 4955–4961.

45. Y. Zhang, C. Wang, N. Wan, Z. Liu and Z. Mao, *Electrochem. Commun.*, 2007, **9**, 667–670.

46. A. Marshall, B. Børresen, G. Hagen, M. Tsypkin and R. Tunold, *Electrochim. Acta*, 2006, **51**, 3161–3167.
